# Artesunate alleviates 5-fluorouracil-induced intestinal damage by suppressing cellular senescence and enhances its antitumor activity

**DOI:** 10.1007/s12672-023-00747-7

**Published:** 2023-07-27

**Authors:** Jing Xia, Qian long Dai, Siyue He, Hui-jie Jia, Xian-Guo Liu, Hui Hua, Min Zhou, Xiaobo Wang

**Affiliations:** 1 School of Basic Medicine, Dali University, Dali, 671000 Yunnan China; 2 Key Laboratory of University Cell Biology Yunnan Province, Dali, 671000 Yunnan China; 3 Department of Oncology, The Affiliated Chengdu 363 Hospital of Southwest Medical University, No. 108, Daosangshu Street, Chengdu, 610041 China

**Keywords:** Artesunate, 5-fluorouracil, Senescence, Intestinal damage, Inflammation, Colorectal cancer

## Abstract

**Background:**

Colorectal cancer (CRC) is one of the most prevalent diagnosed malignancies and one of the leading causes of cancer-related deaths worldwide. 5-Fluorouracil (5-FU) and its combination regimen are commonly used as primary chemotherapeutic agents for advanced CRC. Intestinal mucositis is one of the most frequent side effects of 5-FU. Artesunate (Arte) is derived from the wormwood plant Artemisia annua. Arte is not only effective against malaria but also diabetes, atherosclerosis, inflammation, and other conditions. The mechanism by which 5-FU damages the intestinal tract is unclear, and there is no standard treatment for diarrhea caused by 5-FU. Therefore, it is critical to discover novel and promising therapeutic drugs for 5-FU side effect treatment.

**Methods:**

The morphology and expression of genes and proteins associated with the aging of HUVECs, HIECs, and intestinal tissues were compared to the those of the control group. The cell lines and tissues were evaluated by SA-β-Gal staining, Western blotting, and RT‒qPCR. HIEC and HCT116 cell viability was assessed in vitro by a CCK-8 assay and in vivo by a subcutaneous tumor mouse assay. Tumor cell proliferation and apoptosis was evaluated by immunohistochemistry.

**Results:**

Here, we report that Arte alleviates the adverse side effects caused by 5-FU in intestinal tissue, and that 5-FU-induced intestinal damage is associated with drug-induced chemical inflammation and an increase in the proportion of senescent cells. Arte decreases the ratio of SA-β-Gal-positive cells and downregulated the expression of aging-related proteins (p53, p16) and aging-related genes (p53, p21). Mechanistically, Arte relieves intestinal injury by inhibiting mTOR expression, which is associated with the regulation of aging. Moreover, Arte suppresses the p38MAPK and NF-κB signaling pathways, which are related to inflammation regulation. In addition, the combined therapy of Arte plus 5-FU significantly decreases cancer cell viability in vitro. Arte and 5-FU synergistically reduce the growth of colorectal cancer (CRC) xenografts in vivo.

**Conclusions:**

Overall, our findings point to the crucial treatment effect of Arte on inflammation, intestinal cell senescence, and CRC cell proliferation and offer a new option for CRC treatment.

## Introduction

Colorectal cancer (CRC) is one of the most frequently diagnosed malignancies and the second leading cause of cancer-related mortality worldwide [1]. 5-FU, alone or in combination with other therapeutic drugs, has been used as a standard treatment for CRC for decades. Even though survival rates have improved, 5-FU treatment is highly toxic (leading to side effects such as fatigue, loss of appetite, diarrhea, and neutropenia), has low efficacy, and requires a prolonged treatment course [2]. Hence, it is vital to understand the mechanisms by which 5-FU side effects occur in CRC patients receiving 5-FU treatment. There are multiple mechanisms that underly the pathogenesis of 5-FU-induced colonic mucositis (FUICM), involving the production of ROS, the generation of proinflammatory cytokines, and the infiltration of neutrophils [3]. The pathologies and mechanisms, however, are not entirely understood. Previous research has revealed the pathogenic pathways causing 5-FU-induced cardiotoxicity, including oxidative stress, vascular endothelial dysfunction, extracardiac arterial vasospasm, and apoptosis [4, 5]. Min Zheng. et al. reported that the primary risk element contributing to vascular function disorder and disease progression is endothelial cell senescence. 5-FU can induce endothelial cell senescence [6]. Two recent studies have also shown that removing chemotherapy-induced senescent cells reduces the adverse effects of chemotherapy in mice and improves tissue homeostasis [7, 8]. Therefore, it is necessary to develop drugs that alleviate the cellular aging caused by 5-FU and reduce its side effects.

Cellular senescence is a persistent state of cell cycle arrest that supports tissue remodeling during development and after injury. It can also cause a reduction in tissue regeneration ability, which can lead to loss of function, inflammation, skin damage, and cancer in older people [9, 10]. Senescent cells display considerable morphological alterations, including size differences, irregular shapes,prominent nuclei, and the accumulation of mitochondria and lysosomes. Senescent cells secrete proinflammatory factors, which are collectively referred to as the senescence-associated secretory phenotype (SASP). Researchers have found that the AMPK and mTOR signaling pathways regulate cellular metabolism to preserve energy homeostasis and may also be crucial in the aging process [11, 12]. Researchers have also found that inhibiting the mTOR signaling pathway prolongs the lifespan of cells. AMP-activating protein kinase (AMPK) is a mammalian metabolic sensor that is activated when ATP levels decline in cells [13].

Arte has a variety of properties, including antimalarial, antidiabetic, antiatherosclerotic, and anti-inflammatory properties [14]. Several studies have shown that Arte also has anticancer properties by inhibiting proliferation, invading cancer cells and suppressing angiogenesis [15, 16]. Furthermore,mounting evidence also shows that Arte is effective at sensitizing cells to radiotherapy with X-rays, as well as chemotherapy with cisplatin, doxorubicin, and carboplatin [17]. Jia, H. J. et.al has reported that Arte reduces irinotecan-induced colitis [18]. Whether Arte can alleviate the intestinal damage caused by fluorouracil is unknown. There is no literature reporting the combined anti-inflammatory and antitumor effects of Arte and 5-FU. Based on the above research, we explored the potential effect of Arte on 5-FU therapy in animal models.

Considering the unmet clinical need for therapies that reduce 5-FU-induced intestinal mucositis, we studied the treatment of Arte. In our study, Arte alleviated cellular senescence in HUVECs and HIECs caused by oxidative stress of 5-FU. Arte decreased the ratio in SA-β-Gal-positive cells, the expression levels of aging-related proteins (p53, p16) and genes (p21), the expression levels of SASP factors (IL-1β, IL-6, TNF-α), the protein expression levels of mTOR and NF-κB, as well as the activation of the MAPK signal pathway caused by 5-FU. In the BALB/c mouse model, Atre alleviated intestinal damage caused by 5-FU through antiaging and anti-inflammation effects. In addition, Arte and 5-FU present a cooperative effect on preventing the growth of tumors in CRC xenograft nude mice.

In summary, Arte inhibited 5-FU-induced senescence in HUVEC and HIEC cell models in the current study. The effects of Arte are significant in relieving diarrhea by reducing the accumulation of senescent cells and intestinal inflammation. In addition, Arte has intestinal protective effects associated with cellular aging. Moreover, Arte enhances the antitumor effectiveness of 5-FU treatment. This study provides a new theoretical basis for clinically treating cancer with Arte.

## Materials and methods

### Cells lines and treatments

Human umbilical vein endothelial cells (HUVECs) were obtained from the Institute of Biological Sciences, Chinese Academy of Sciences (Shanghai). HUVECs were kept in RPMI-1640 medium from Invitrogen in Gaithersburg, Maryland, which also contained 10% fetal bovine serum, 2 mM l-glutamine, 100 U/mL penicillin, and 100 mg/mL streptomycin (complete medium). Human intestinal epithelial cells (HIECs) were supplied by Guangzhou Jennio Biotech Co., Ltd. Both HIEC and HCT116 (ATCC #CCL-247) cell lines were cultured in DMEM (Dulbecco’s modified Eagle’s medium, Thermo Fisher Scientific) containing 10% FBS at 37 °C in a humidified environment consisting of 95% air and 5% CO_2_. All cell lines were passaged for fewer than 6 months after being revived. Artesunate (S2265), 5-fluorouracil (S1209), and insulin (S6955) were supplied by Selleck. H_2_O_2_ (10011208) was purchased from Wokai Biotechnology (Shanghai, China). They were reconstituted in dimethyl sulfoxide (DMSO) and kept at 20 °C for each experiment. The ultimate 5-FU concentration employed was 10 mmol/L in all experiments.

### Animals

Adult male BALB/c and BALB/c nude mice (6–8 weeks old, 20–25 g, Kunming, China) were placed into a routine laboratory environment (12-h cycle of light and dark, 22 ± 2 °C temperature). Upon approval by the Ethics and Protection of Animals Committee of Dali University (Number: 2022-P2-21, Dali, Yunnan, China), the experimental protocols were implemented.

### Reagents and antibodies

Anti-GAPDH (ab8245, 1:2000) was obtained from Abcam. Rabbit anti-phospho-mTOR (Ser2448) polyclonal antibody (Ser2448) (#2971, 1:1000), anti-p38MAPK (#6417S, 1:1000), anti-phospho-AMPKα (Thr172) (#2535, 1:1000), and phospho-NF-κB p65 (Ser536) (7F1) mouse mAb (Cat#3036, 1:1000) were obtained from CST (Cell Signaling Technology). anti-ki-67 (GB111499, 1:800), PCNA (GB11010-1, 1:1500), caspase-3 (GB11009-1, 1:200), and anti-Bcl-2 rabbit pAb (GB113375-1, 1:200) were purchased from Servicebio (Wuhan, China). anti-NF-κB p65 recombinant rabbit monoclonal antibody [SZ10-04] (ET1603-12, 1:1000), anti-p38 alpha/MAPK14 recombinant rabbit monoclonal antibody [SR43-04] (ET1602-26, 1:1000), anti-mTOR recombinant rabbit monoclonal antibody [SU30-00] (ET1608-5, 1:1000), and anti-p16 recombinant rabbit monoclonal antibody [PD01-16] (ET1602-9, 1:1000) were purchased from HuaBio (Hangzhou, China). p53 polyclonal antibody (10442-1-AP, 1:1000) was purchased from Proteintech (Wuhan, China). anti-PRKAB1 rabbit polyclonal antibodies (D221614, 1:1000) were purchased from Sangon Biotech (Shanghai China). anti-mouse IgG HRP (horseradish peroxidase)-linked antibody (H + L) (7076the P2, 1:15000) was purchased from CST, and HRP-linked rabbit IgG antibodies were purchased from CST. Reagents for enhanced chemiluminescence (ECL) were purchased from Bio-Rad (USA).

### Cell viability assay

The Cell Counting Kit-8 reagent (CCK-8 PF00004) was purchased from ProteinTech (Beijing, China). The cells were grown in 96-well dishes at a density of 1.0 × 10^4^ cells /100 µL/well and then incubated for 96 h with 5-FU (1 µM) or 5-FU plus Arte at different concentrations (2–15 µM). In 96-well plates, CCK8 reagent was added two hours before harvesting, and the density of absorbance at 450 nm was determined by a plate reader (five wells per concentration). The cell viability ratio was calculated as follows: Cell viability ratio = [(As − Ab)/(Ac − Ab)] × 100%. As: experimental group absorbance (containing cell, DMEM, CCK-8 solution, drug solution); Ac: control group absorbance; Ab: blank group absorbance.

### SA-β-Gal staining

Positively stained cells for SA-β-Gal are considered to be a common indicator of cellular senescence [19]. SA-β-Gal staining was performed using an SA-β-Gal staining kit (Solarbio) to assess the senescence of HUVECs, HIECs and frozen intestinal sections (4 µm). To screen for antioxidative stress drugs, after H_2_O_2_ exposure for 2 h, HUVECs in combination with Arte (0.5–6.0 µM) were cultured for another 4 days. Similarly, 5 days after 5-FU plus Arte (2–15 µM) exposure, HUVECs and HIECs were grown in a 12-well plate and incubated at 80% confluence. After fixing the cells for 10 min at room temperature, they were washed three times with PBS before being stained with a working solution at 37 °C for 12 h. Images were captured with a light microscope. The cell images were captured at a magnification of 100 ×; the animal tissue images were captured at magnifications of 100 × and 400 ×.

### Western blotting

The cells and intestinal tissues were lysed by using phosphatase (Sigma‒Aldrich) and protease (Roche) inhibitors in RIPA buffer (Pierce). The protein concentration was determined using the BCA method (Solarbio, China), and 2.5 × SDS loading buffer was added to the lysates following 10 min of boiling. The same amount of protein was separated by SDS‒PAGE and transferred to polyvinylidene difluoride (PVDF) membranes for each sample. Positive bands were visible using enhanced chemiluminescence detection reagents (Santa Cruz) after probing with the primary and horseradish peroxidase-conjugated secondary antibodies.

### Real-time quantitative PCR

TRIzol reagent (Invitrogen) was used to extract intestinal tissue, and 5 × 10^6^ cells and 1 mg of each sample were reverse transcribed using a PrimeScript RT reagent kit (Cat#TSK322S, Tsingke, Beijing, China). The reverse transcription conditions were as follows: 25 °C for 10 min → 50 °C for 15 min → 85 °C for 5 MIN. Fifty nanograms of total cDNA was amplified using 2 × TSINGKE^R^Master qPCR (SYBR Green) (Tsingke, Beijing, China). The comparative CT approach was used to examine relative gene expression, with 18S ribosomal RNA serving as the housekeeping gene. The following primers are provided in Table 1.

**Table 1:** Primers used in this study

Gene	Sequence (5′–3′)
h-p53 F:GAGGTTGGCTCTGACTGTACC 21 bp	R:TCCGTCCCAGTAGATTACCAC 21 bp
h-p21 F: TGTCCGTCAGAACCCATGC 19 bp	R: AAAGTCGAAGTTCCATCGCTC 21 bp
h-IL-1β F: ATGATGGCTTATTACAGTGGCAA 23 bp	R: GTCGGAGATTCGTAGCTGGA 20 bp
h-IL-6 F: ACTCACCTCTTCAGAACGAATTG 23 bp	R: CCATCTTTGGAAGGTTCAGGTTG 23 bp
h-TNF-α F: CCTCTCTCTAATCAGCCCTCTG 22 bp	R: GAGGACCTGGGAGTAGATGAG 21 bp
m-p21 F: GTGGCCTTGTCGCTGTCTT 19 bp	R: GCGCTTGGAGTGATAGAAATCTG 23 bp
m-IL-1β F: TGGACCTTCCAGGATGAGGACA 22 bp	R: TGGACCTTCCAGGATGAGGACA 22 bp
m-IL-6 F: ACTCACCTCTTCAGAACGAATTG 23 bp	R: CCATCTTTGGAAGGTTCAGGTTG 23 bp
m-TNF-α F:CAAGATGATCTGAGTGTGAGGGT 23 bp	R:GCAATACGGACTTGCTCACAGA 22 bp
m-p53 F: TGCGTGTGGAGTATTTGGATG 21 bp	R: TGGTACAGTCAGAGCCAACCTC 22 bp
m-p21 F: GTGGCCTTGTCGCTGTCTT 19 bp	R: GCGCTTGGAGTGATAGAAATCTG 23 bp
m-18 s F:TTGACGGAAGGGCACCACCAG 21 bp	R: GCACCACCACCCACGGAATCG 21 bp

The data were analyzed using Step One Software (v2.2, Waltham, Mass, USA) to obtain the relative expression of genes. The 2^*−*△△CT^ method was used to calculate the expression of each gene and gene expression was normalized to that of 18S

### Mucositis induction and treatment protocol

In BALB/c mice, 5-FU (50 mg/kg) was intraperitoneally (i.p.) administered every two days for a total of four days (Days 2, 4, 6, 8), and the control group (CTL) received 0.9% sodium chloride (NaCl). Arte (30 mg/kg) was intraperitoneally administered intraperitoneally once daily, and PBS (the vehicle for artesunate) was given to the control group for 9 days (Day 1 to Day 9). The 5-FU dose was calculated according to our previous literature [20]. The mice were weighed every day, and the quality of their feces was used to gauge the severity of the disease. According to Hiroyasu Sakai et al. [21], the stools were categorized as previously described. 1: normal; 2: mild diarrhea (squishy stool); 3: moderate diarrhea (unformed, somewhat watery stool); and 4: extreme diarrhea (watery stool and noticeable perianal stains). Animals were sacrificed one day after the last Arte injection (Day 10). Mouse intestinal tissues (colon and ileum) were gathered, and one of the samples of intestinal tissue was submerged in 4% paraformaldehyde. For staining with eosin + hematoxylin (H + E) (Solarbio, China), the paraffin-fixed intestinal tissue samples were cut into 4 µm thick sections. Images were collected using a microscopy imaging system (AxioScope. A1, Germany). A second portion of the tissue sample was embedded in optimal cutting temperature compound and then sliced into 8 µm-thick sections for SA-Gal staining.

### Xenograft model

BALB/c nude mice (male, 6–8 weeks old, weighing 20–25 g) were provided by the Kunming Institute of Zoology, Chinese Academy of Sciences. HCT116 cells (10 × 10^6^) were subcutaneously inoculated into the left thigh dorsal region of nude mice, and tumor size was measured every 2 days for xenograft model establishment. After 7 days of inoculation with a tumor size of approximately 100 mm^3^, the vehicle (200 µL 0.9% NaCl) was intraperitoneally delivered into the mice, 5-FU (30 mg/kg thrice a week), Arte, 5-FU + Arte (30 mg/kg every day) for 14 days. The tumor size did not exceed the maximum requirements of the Ethics Committee of Dali University. When the tumor in the control group reached 1000–2000 mm^3^, the tumor volumes were calculated using the following formula [22]: V = a*b^2^/2, where a represents the largest tumor diameter in millimeters and b represents the shortest tumor diameter. The tumors were isolated for the following experiments.

### HE staining

HE staining of intestinal tissue and tumor paraffin-embedded sections was performed as directed (Solarbio). Sections were washed with PBS three times. The intestinal tissue sections were washed three times with PBS, stained with hematoxylin solution for 1 min, differentiated, and then stained with eosin solution for 1 min. The tumor sections were stained with hematoxylin solution for 5 min, differentiated, and then stained with eosin solution for 5 min. Images of the tissue were taken using a light microscope (n = 6).

### Immunohistochemistry

As previously described previously, xenograft tumors were formalin-fixed, paraffin-embedded, and sectioned [23]. We used a MaxVision kit to stain the sections (Maixin Biol, Fuzhou, Fujian, China) according to the manufacturer's instructions. Fifty microliters of MaxVision TM reagent was employed in addition to the primary antibodies on each slide. Then, 0.05% diaminobenzidine and 0.03% H_2_O_2_ in 50 mM Tris–HCl were used for color development (pH 7.6), and hematoxylin was used as a counterstain on the sections. The negative control was preimmune rabbit serum.

### Statistical analyses

The data are presented as the mean ± standard deviation of at least 3 separate experiments. Statistical analysis was conducted using GraphPad Prism (Version 6.0) software. One-way ANOVA analyzed the differences among several groups with Tukey's post hoc test. *p* values < 0.05 were deemed statistically significant.

## Results

### Artesunate suppresses oxidative stress-induced aging in HUVECs

**Fig. 1:**
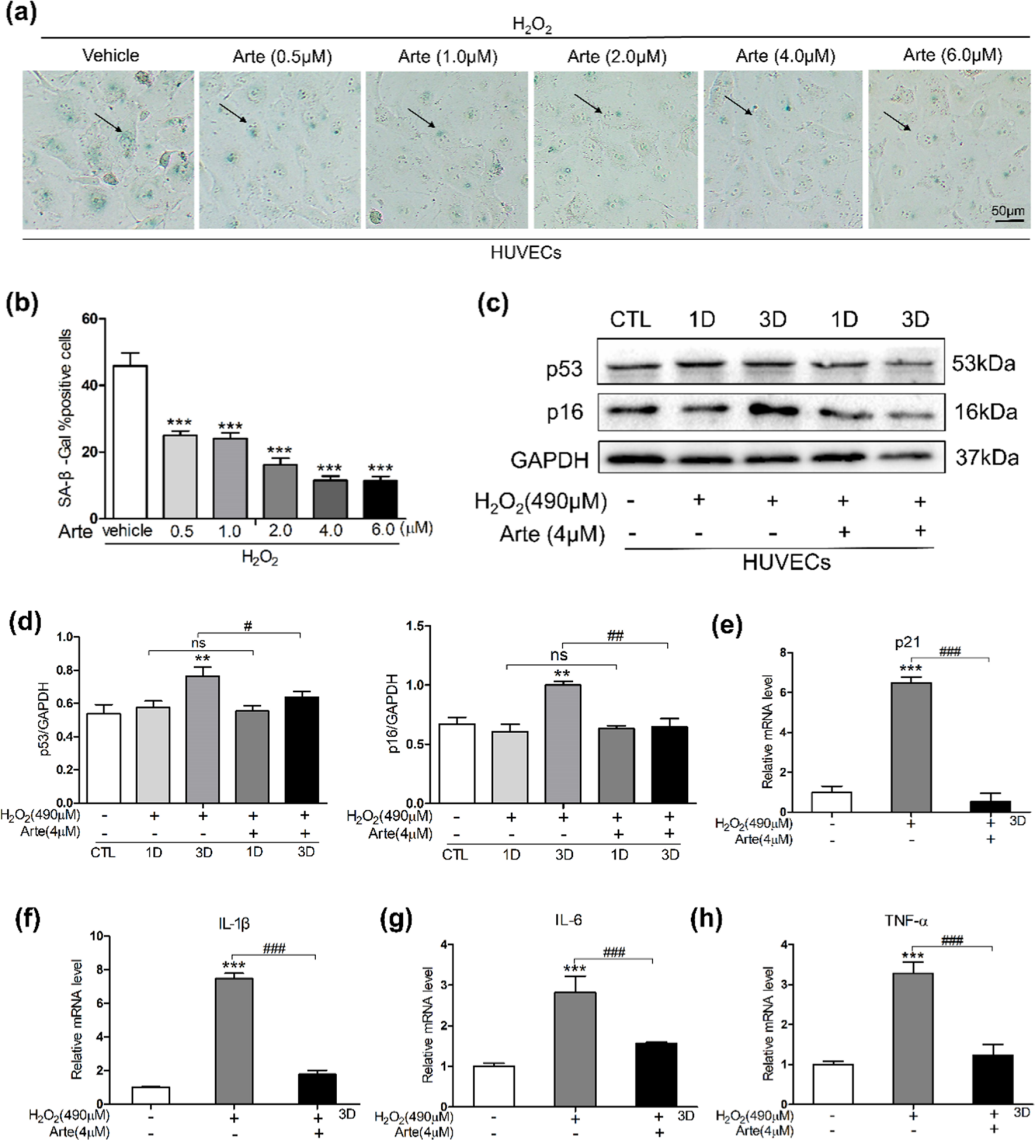
Artesunate suppresses oxidative stress-induced aging in HUVECs. **a**, **b** Images and percentages of SA-Gal-positive HUVECs treated with 490 µM H_2_O_2_ plus various Arte concentrations. Scale bar: 50 μm. **c** p53, p16, and GAPDH protein levels of HUVECs treated with H_2_O_2_ on Days 1 and 3 (**d**) Relative protein level ratio of p53, p16, and GAPDH. **e**–**h** mRNA expression of p21, IL-1β, IL-6, and TNF-α of H_2_O_2_ and in combination with Arte were measured by RT‒qPCR. *p < 0.05, **p < 0.01, ***p < 0.001 compared to the control group (CTL); #p < 0.05, ##p < 0.01, ###p < 0.001 compared to the vehicle control or the indicated sample

HUVECs were treated with H_2_O_2_ for 2 h. Then, different concentrations of Arte were added to H_2_O_2_-treated HUVECs and coincubated for 5 days for SA-β-Gal staining, as shown in Fig. 1a. The proportion of senescent cells was determined by SA-β-Gal staining. Figure 1a, b shows that the ratio of SA-β-gal-positive cells in HUVECs after H_2_O_2_ treatment was significantly increased to approximately 40–50%; however, in the presence of Arte, the ratio of SA-β-Gal-positive cells significantly decreased. We selected the concentration of 4 µM Arte for subsequent experiments. To demonstrate the antiaging effect of Arte, we conducted Western blotting and RT‒qPCR experiments. Protein samples were collected on Days 1 and 3 from H_2_O_2_-treated and H_2_O_2_ + Arte cotreated HUVECs (Fig. 1c, d). The protein levels of p16 and p53 were slightly higher on Day 1 after H_2_O_2_ treatment, but there was no statistically significant difference compared to those on Day 1 in the Arte-treated group. The levels of p53 and p16 protein were significantly increased at Day 3 after H_2_O_2_ exposure, while Arte treatment reversed these effects. We believed that oxidative stress-induced changes mainly occurred on the third day after cells were stimulated by external factors, so we collected HUVECs from different treatment groups on the third day. Next, we performed age-related genetic (p21, IL-1β, IL-6, TNF-α) testing on different groups of HUVECs. As shown in Fig. 1e–h, compared with the control group, the levels of p21, IL-1β, IL6, and TNF-α increased significantly after H_2_O_2_ treatment, while the levels of aging-related genes obviously decreased after Arte treatment. Through SA-β-Gal staining experiments, Western blotting, and RT‒qPCR experiments, it was proven that oxidative stress can accelerate HUVEC aging, and Arte can ameliorate this process.

### Artesunate alleviates the senescence of HUVECs and HIECs induced by 5-FU

**Fig. 2:**
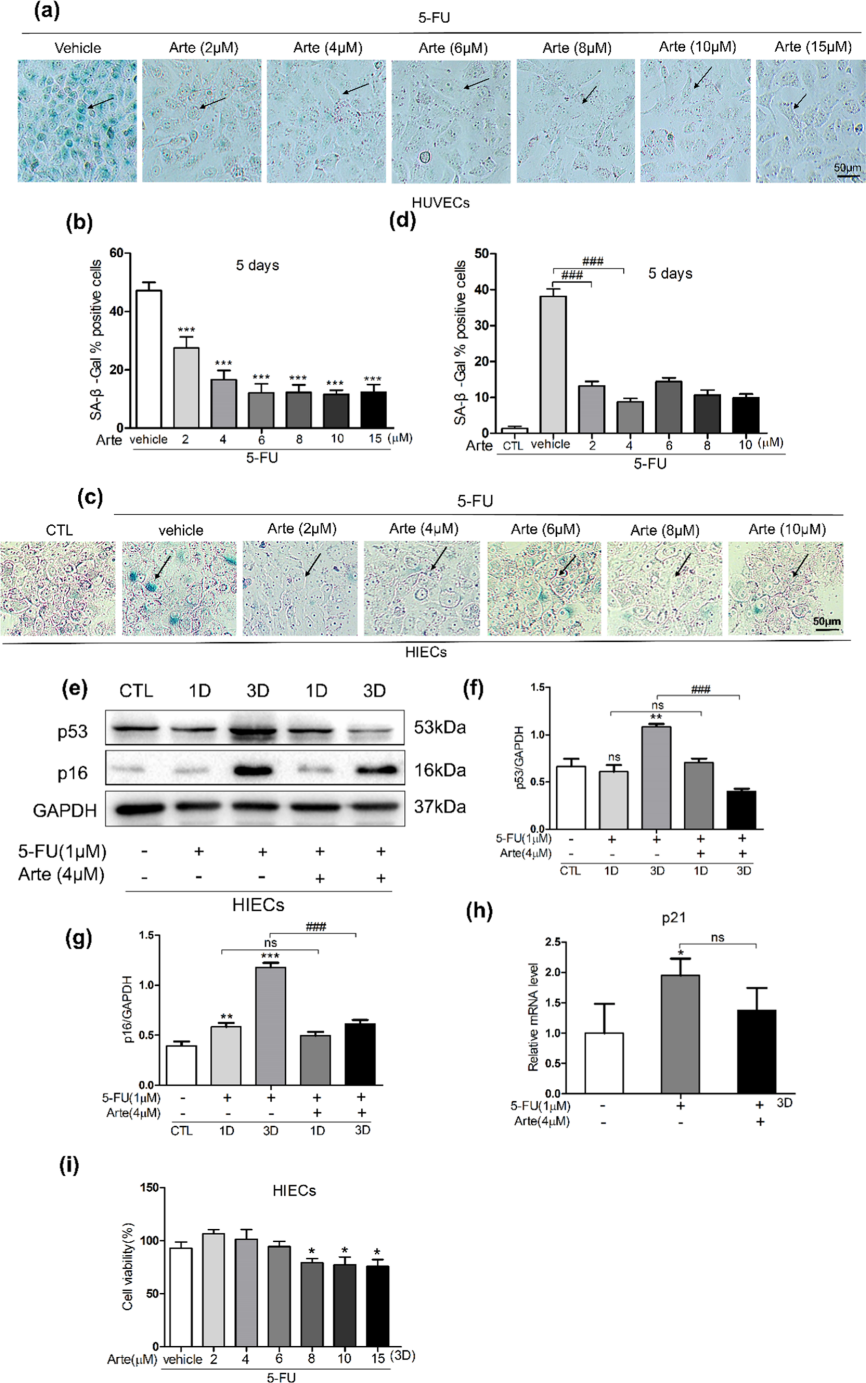
Arte alleviates 5-FU-induced HUVEC and HIEC senescence. Cells were incubated with different concentrations of Arte and 1 µM 5-FU for 5 days and then were subjected to SA-β-Gal staining. **a**, **b** Representative images and the proportion of SA-β-Gal-positive HUVECs scale bars: 50 μm; **c**, **d** Representative images and proportions of SA-β-Gal-positive HIECs, scale bar: 50 μm; **e** Images of Western blotting of p53, p16, and GAPDH; **f**, **g** Relative protein level ratio of p53, p16, and GAPDH, GAPDH was used as loading control; **h** The expression of p21 mRNA of 5-FU and Arte (4 µM) treated HIECs, respectively; (i) The cell viability of HIEC was evaluated by a CCK-8 assay; *p < 0.05, **p < 0.01, ***p < 0.001 compared to the control group (CTL); #p < 0.05, ##p < 0.01, ###p < 0.001 compared to the vehicle control or the indicated sample. 1D-day one; 3D-day three

In our experiment, Arte significantly reduced the proportion of senescent cells in HUVECs caused by 5-FU from 50 to 10%, especially if the concentration of Arte was between 4 and 10 µM (Fig. 2a, b). 5-FU is the most commonly used chemotherapeutic agent, and one of its common side effects is gastrointestinal damage. We speculated that the adverse reactions of the digestive tract caused by 5-FU might be related to the induction of intestinal epithelial cell senescence and the chemical inflammation of digestive tract mucosa caused by chemotherapy drugs. To test our hypothesis, we used HIEC cells for related experiments. According to the preliminary results, 1 μM 5-FU was selected to treat HIECs. 5-FU caused senescence in HIECs in accordance with the hypothesis. In comparison to control cells, HIECs showed large and flat positively-stained cells after 5-FU treatment. We cocultured cells with 1 μM 5-FU and various concentrations of Arte for 5 days and then stained for SA-β-Gal to evaluate the anti-aging effect, especially at doses of 2 to 4 μM. Arte significantly reduced the proportion of SA-β-Gal-positive cells (Fig. 2c, d). In addition to Western blotting for detecting aging-related proteins, RT‒qPCR was used to measure the expression of p21gene associated with aging. Arte significantly reduced the protein expression levels of p53 and p16 and the mRNA expression level of p21 (Fig. 2e–h). Furthermore, HIEC viability after Arte exposure was investigated. Figure 2i demonstrates that the two-drug combination had no impact on cell viability when the concentration of Arte was less than 6 µM. However, there was a statistically significant decrease in the cell viability of HIECs when the concentration of Arte was higher than 8 μM. In conclusion, HUVEC and HIEC senescence is induced by 5-FU, and Arte can delay aging.

### In the BALB/c mouse model, Arte can alleviate intestinal damage and aging caused by 5-FU

**Fig. 3:**
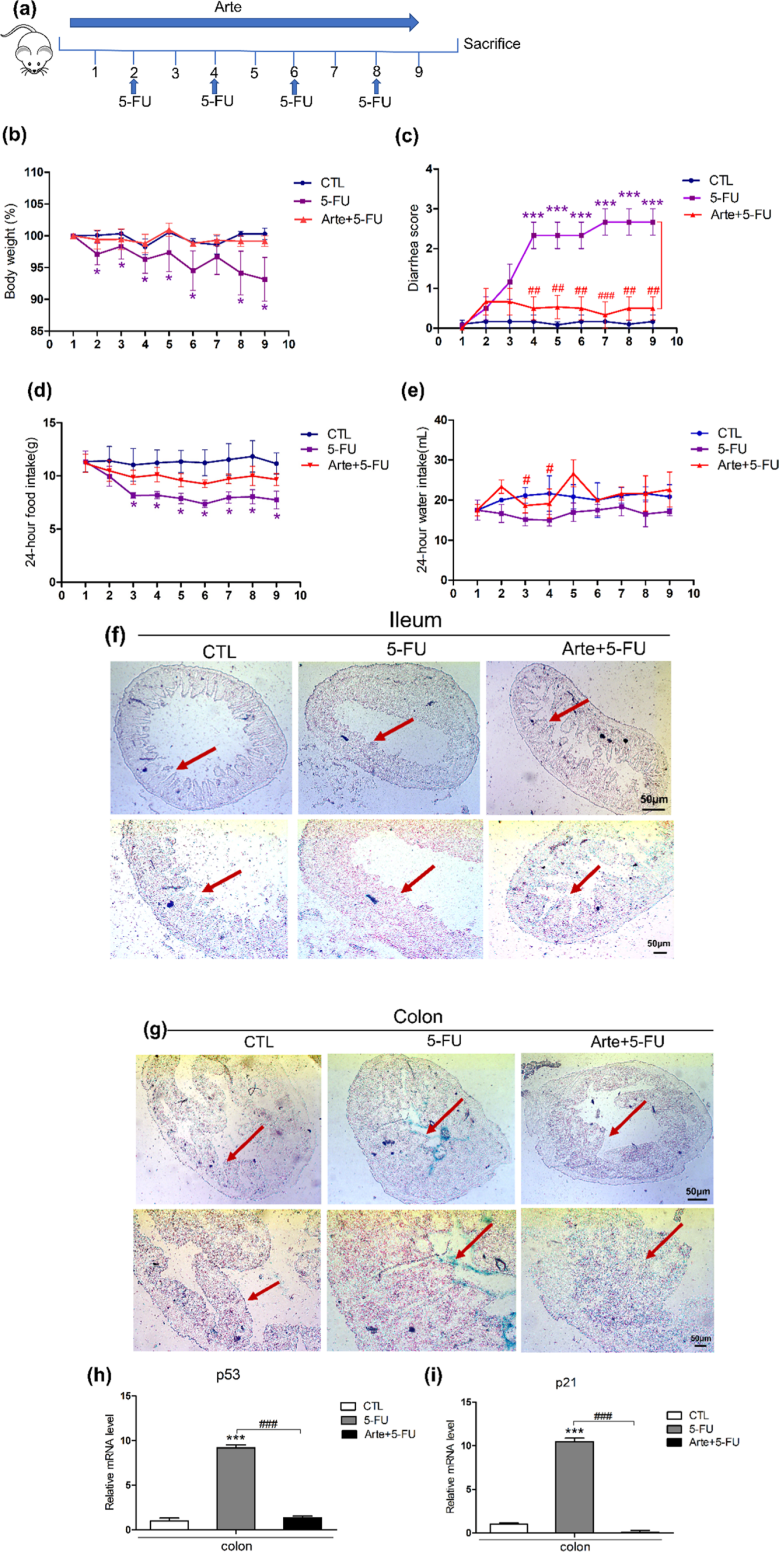
Arte attenuates 5-FU-induced intestinal damage in BALB/c mice. **a** The establishment of the diarrhea model and drug implementation. **b** Weight curve of mice in three groups (control, 5-FU, 5-FU + Arte); **c** diarrhea index of mice in three groups; **d** 24-h food intake of mice in three groups; **e** Each group of mice at 24-h water intake; **f**, **g** SA-β-Gal staining of each group of tissues (ileum, colon); Scale bars: 50 μm; **h**, **i** mRNA expression levels of p53 and p21 in colonic tissue. *p < 0.05, **p < 0.01, ***p < 0.001 compared to the control group (CTL). #p < 0.05, ##p < 0.01, ### p < 0.001 compared to the vehicle control or the indicated sample. 1D-day one; 3D-day three

Figure 3a shows the establishment of the diarrhea model and drug implementation. We evaluated the effect and safety of Arte in improving 5-FU-induced intestinal injury in a mouse model of BALB/c. The weights of mice receiving 5-FU at 50 mg/kg were consistently lower than those in the control group mice, while body weight was improved in the Arte-treated group mice (control 23.75 ± 1.81 g, 5-FU 20.6 ± 3.17 g, Arte + 5-FU 22.52 ± 1.8 g) (Fig. 3b). As shown in Fig. 3c, 5-FU-treated mice developed diarrhea of different degrees starting from the second day. Atre treatment decreased the 5-FU-induced diarrhea score (Fig. 3c). The 24-h food intake of the 5-FU treatment group demonstrated a statistically significant decrease, and the food intake of the combined treatment group was improved (control 11.57 ± 1.9 g/d, 5-FU 8.21 ± 1.01 g/d, Arte + 5-FU 10.28 ± 1.05 g/d) (Fig. 3d). Examining the 24-h water intake per mouse, Arte improved the 5-FU-induced decline in water intake (Fig. 3e). Next, to investigate intestinal cell aging, cryosections of ileum and colon tissue from the control, 5-FU, and 5-FU + Arte groups were stained with SA-β-Gal. In the ileum of 5-FU-treated mice, no SA-β-Gal-positive villi were observed, and the villi were shorter and thinner. However, in the colon tissue, colonic frozen sections were positively stained after 4 h of immersion in SA-β-Gal staining. These results indicate that 5-FU treatment can damage the colonic mucosal epithelium and destroy the villi structure. In contrast, Arte reduced mucosal damage (Fig. 3f, g). We further examined the expression of the genes p53 and p21 associated with aging in colon tissue, and then the RNA was extracted for RT‒qPCR. In the 5-FU-treated group, the expression of the age-related genes p53 and p21 was markedly upregulated, while Arte obviously downregulated the expression of p53 and p21 (Fig. 3h, i). These findings suggest that Arte can alleviate intestinal damage and senescence caused by 5-FU.

### Arte alleviates aging by inhibiting the mTOR signaling pathway in vitro and in vivo

**Fig. 4:**
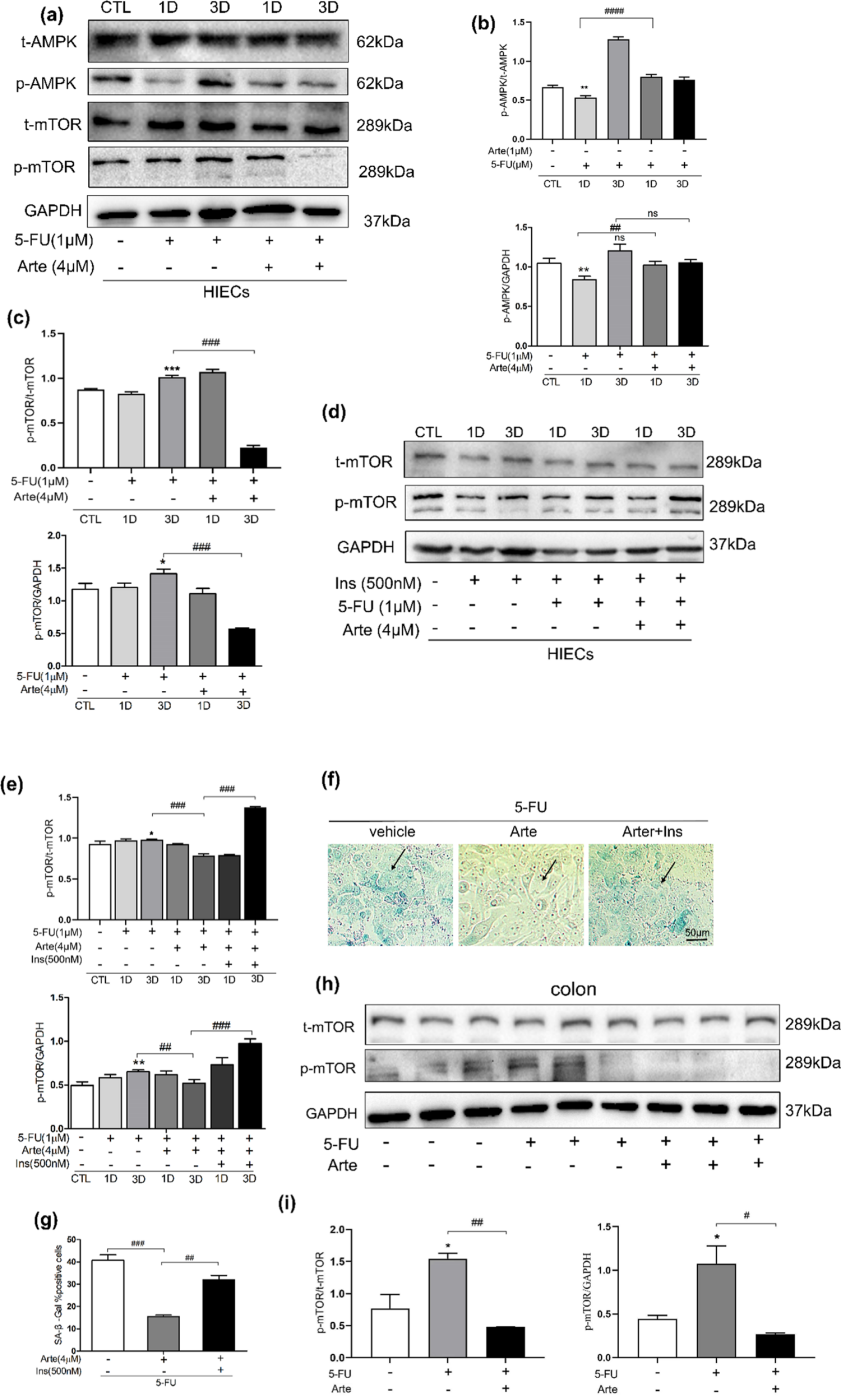
Arte delays cellular aging by inhibiting the mTOR pathway in HIEC and BALB/c mouse models. **a** HIEC treated with 5-FU (1.0 μM) or 5-FU plus Arte (4 µM) on Days 1 and 3; Representative images of immunoblotting of t-mTOR, p-mTOR, t-AMPK, p-AMPK, and GAPDH; (**b**, **c**) Quantification of relative protein levels (n = 6); **d** Representative images from t-mTOR, p-mTOR and GAPDH Western blot assays; **e** Quantification of relative protein levels, n = 6; (**f**, **g**) SA-β-Gal positive representative images and statistical charts of HIEC after different concentration of drug treatment with HIEC, scale bar: 50 μm; (**h**, **i**) The t-mTOR, p-mTOR protein expression in mice with 5-FU-induced diarrhea; Protein samples were taken from colonic tissue of 6 mice in each group and Western blot analyses were performed (n = 6); *p < 0.05, **p < 0.01, ***p < 0.001 compared to the control group (CTL); #p < 0.05, ##p < 0.01, ###p < 0.001 compared to the vehicle control or the indicated sample. 1D-day one; 3D-day three

We evaluated the status of the mTOR and AMPK signaling pathways to determine the mechanism by which Arte inhibits 5-FU-induced endothelial and intestinal senescence. HIEC cells exhibited upregulated expression of the p-mTOR protein after 5-FU treatment on Day 1 and day 3; Arte significantly downregulated the expression of the p-mTOR protein in HIEC cells treated with 5-FU (Fig. 4a–c). As a further demonstration, we conducted an experiment involving activation. With insulin added to HIEC cells, p-mTOR protein levels were significantly increased, and Arte's anti-aging effects were significantly reduced (Fig. 4d, e). In Fig. 4f, g, after insulin was added, the antiaging effect of Arte administration in HIEC cells was eliminated, and a higher proportion of SA-β-Gal senescent cells appeared in the Arte + 5-FU + Ins treatment group. In the BALB/c mouse model, in colonic tissue, 5-FU treatment markedly increased p-mTOR protein levels, whereas Arte substantially decreased p-mTOR protein levels (Fig. 4h, i). These results suggest that Arte can delay aging by inhibiting the mTOR signaling pathway in vivo and in vitro. Additionally, we found that 5-FU increased the levels of p-AMPK protein as the number of treatment days increased (Fig. 4a, b). However, the AMPK phosphorylation levels of Arte did not show a trend in this study, and the underlying mechanisms were not investigated.

### In vivo and in vitro, Arte relieves inflammation by inhibiting the p38MAPK and NF-κB signaling pathways

**Fig. 5:**
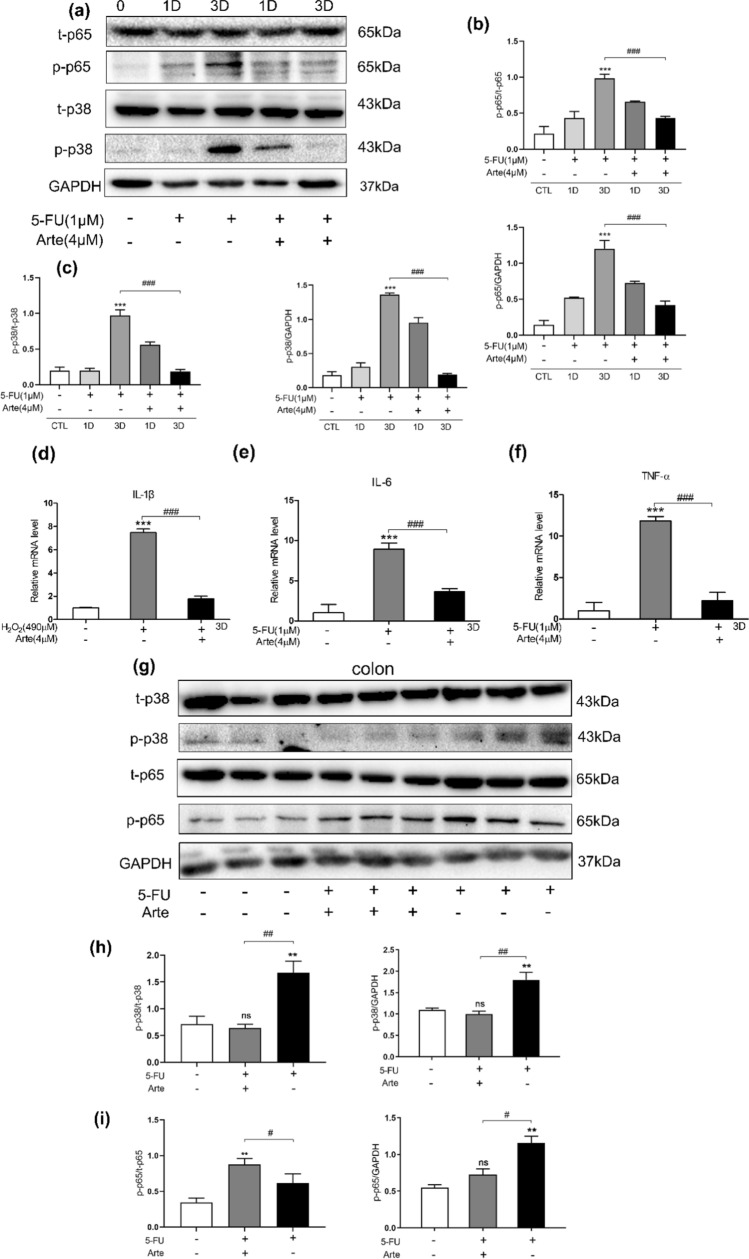

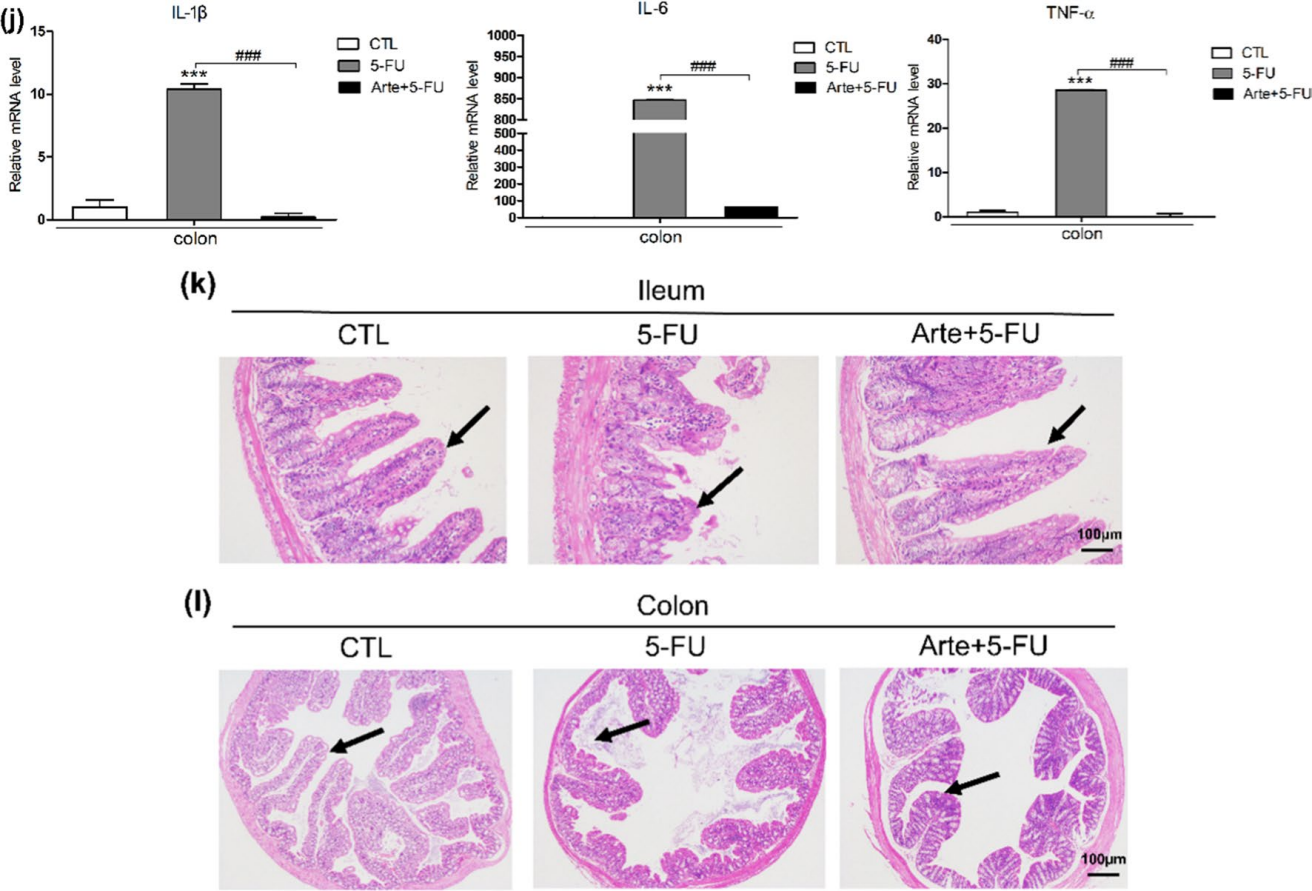
Arte alleviates 5-FU-induced inflammation by inhibiting p38MAPK and NF-κB in cells and tissues. **a** Western blotting of t-p65, p-p65, t-p38, p-p38 and β-actin protein levels in HIEC cells. **b**, **c** Quantification of relative protein levels, n = 6. **d**–**f** Relative gene expression was normalized to that of 18S, and the genes were associated with the p38MAPK/NF-κB signaling pathway, as detected by RT‒qPCR. **g** Image of relative protein levels of t-p65, p-p65, p-p38, t-p38, and β-actin in colonic tissue, n = 6. **h**, **i** Quantification of relative protein levels; **j** Relative gene expression was normalized to that of 18S in colon tissue. **k**, **l** Comparison of histopathological changes in each treatment group by H&E staining (100 × magnification), n = 6. *p < 0.05, **p < 0.01, ***p < 0.001 compared to the control group (CTL); #p < 0.05, ##p < 0.01, ###p < 0.001 compared to the vehicle control or the indicated sample. 1D-day one; 3D-day three

In our study, a Western blotting experiment was conducted to investigate the effect of 5-FU on the phosphorylation levels of p38 and p65. In HIEC cells, the protein expression levels of p-p65 (Fig. 5a, b) and p-p38 (Fig. 5a–c) significantly increased after treatment with 5-FU on Days 1 and 3. Arte reduced the levels of p38 and p65 phosphorylation. Senescent cells produce extracellular albumin and inflammatory cytokines linked to chronic inflammation [34]. The levels of pro-inflammatory cytokines such as IL-1β, TNF-α and IL-6 were determined by RT‒qPCR. Arte alleviates the increased levels of inflammatory cytokines induced by 5-FU on day 3 (Fig. 5d–f). In the intestinal injury BALB/c mouse model, we found that Arte significantly decreased the levels of p-p38 and p-p65 in the colon tissues of mice receiving 5-FU (Fig. 5g–i). The accumulation of proinflammatory cytokines secreted by senescent cells in tissues, namely, SASP factors (IL-1β, IL-6, and TNF-α), significantly increased in the intestinal tract of mice treated with 5-FU. Interestingly, compared with the 5-FU group, the Arte group exhibited a dramatically decreased levels of inflammatory cytokines, which were increased by 5-FU (Fig. 5j). In the control group, the intestinal structure was intact, and the chorionic glands were normal without hyperemia or edema. The submucosa and lamina propria of the mucosa were intact. 5-FU treatment injured the structure of intestinal mucosa, crypt-villus, and mucosal epithelium. In contrast, Arte reduced mucosal damage and regulated the structure of the crypt villi (Fig. 5k, l). The results above indicate that Arte reduces inflammation caused by 5-FU in HIEC cells and animal intestinal injury models via the p38MAPK and NF-κB signaling pathways.

### In vitro and in vivo, Arte enhances the antitumor effect of 5-FU on HCT116 cells

**Fig. 6:**
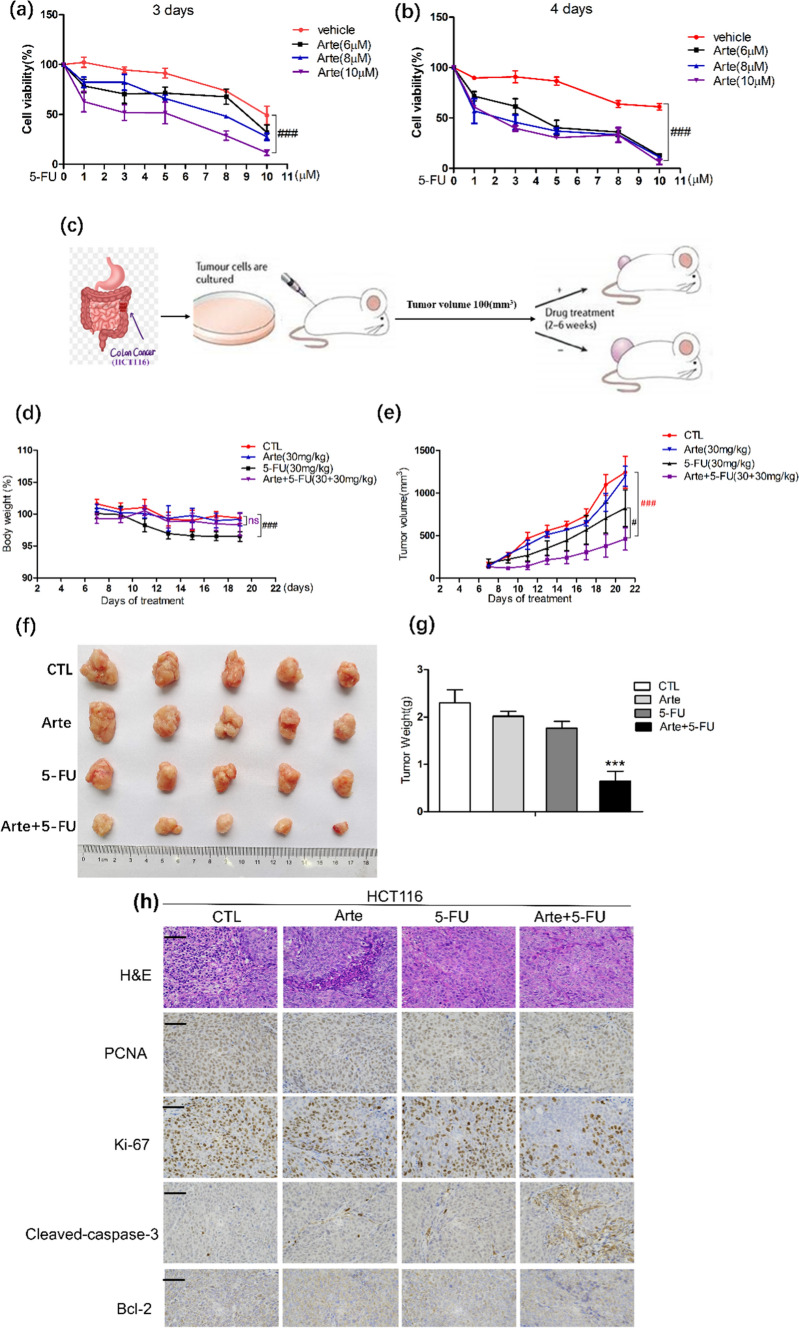

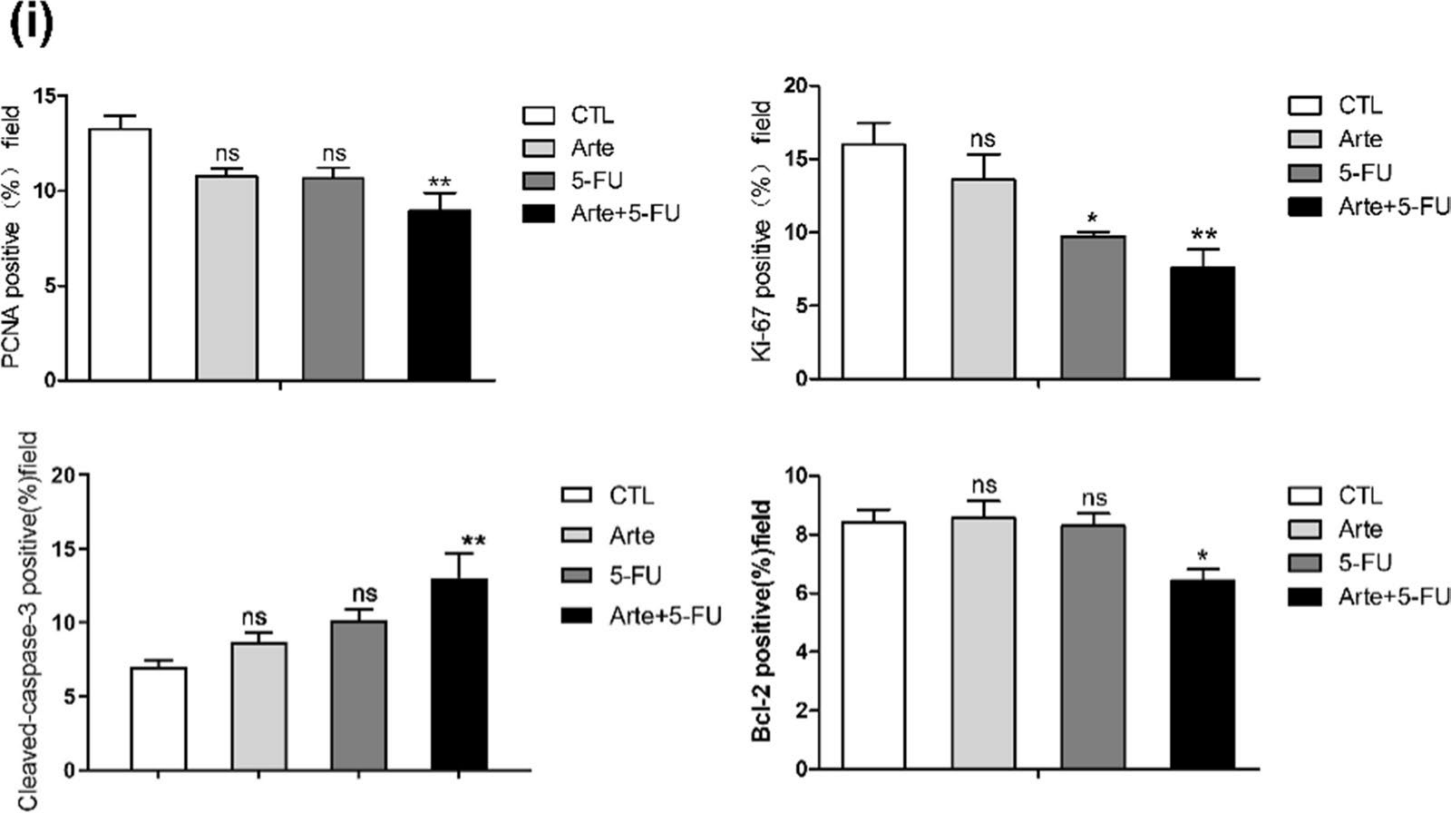
Arte enhances the antitumor effect of 5-FU in vitro and in vivo. **a**, **b** Cell viability of HCT116 cells treated with different concentrations of Arte, 5-FU and the combination on Days 3 and 4. **c** Schematic of animal model experiment. **d** Line graph of changes in body weight during treatment in BALB/c nude mice, n = 6. **e** Line graph of the tumor growth volume. **f** Images of the tumors from each group. **g** The weight of tumors in each treatment group of BALB/c nude mice; **h** HE staining of paraffin-embedded sections (4 μm) of tumor tissue and immunohistochemical assay proliferation refers to labeled PCNA, Ki-67, Cleaved-caspase-3, Bcl-2, scale: 50 μm; (i). Immunohistochemistry analyses. *p < 0.05, **p < 0.01, ***p < 0.001 compared to the control group (CTL); #p < 0.05, ##p < 0.01, ###p < 0.001 compared to the vehicle control or the indicated sample

The HCTT116 cell line was selected to test whether Arte enhances the antitumor sensitivity of 5-FU in vitro, and a CCK-8 assay was performed. When treated with 5-FU alone for 3 days, the survival rate of HCT116 cells was higher than 50% or above when the minimal combination concentration of 5-FU was 8 μM plus 5 μM Arte, and the survival rate of HCT116 cells was lower than 50% on the 4th day, when the lowest concentration of 6 μM Arte plus 5 μM 5-FU resulted in a 40.38% cell survival ratio. However, 5-FU at 5 μM alone showed no significant antitumor effect on HCT116 cells with near-100% viability (Fig. 6a, b). Combined with our study, Arte enhanced the anticancer effect of 5-FU when they were combined. This research result provides a new therapeutic strategy for clinical treatment. Next, we conducted an animal model experiment according to the operation protocols in Fig. 6c. The nude mice in both the treatment and control groups tolerated the drug regardless of whether it was Arte alone or Arte combined with 5-FU, with no significant adverse effects. In all four groups of nude mice, there was no statistically significant weight loss except in the 5-FU-treated group (Fig. 6d). Starting from the 7th day of treatment, the antitumor activity of Arte combined with 5-FU was shown to be higher than that of 5-FU alone, even though 5-FU had some antitumor activity compared to the groups of Arte and control alone (Fig. 6e). The tumor size and weight of the 5-FU group were smaller and lighter than those of the Arte and control groups. However, the combined treatment group showed a more significant antitumor effect (Fig. 6f, g). To further demonstrate the antitumor efficacy of the combination of two drugs, HE staining and immunohistochemical detection of PCNA, Ki-67, Cleaved-caspase-3, and Bcl-2 were performed on the tumor tissues. As shown in Fig. 6h, i, HE staining showed that compared to the control group and the Arte group, the 5-FU group exhibited tumor cells that appeared necrotic, and the Arte + 5-FU group exhibited necrotic cells and a large number of vacuoles. PCNA is intimately connected to DNA synthesis, which is crucial for the start of cell proliferation. The tumor tissue proliferation indicators PCNA and Ki-67 reflect the proliferation status of cells. As described in the literature [24], PCNA and Ki-67 localized in the nucleus and showed brownish-yellow staining, and brownish-yellow staining significantly increased during tumor expansion. The proportion of PCNA- and Ki-67-positive tumor cells in the control group significantly increased compared with that in the Arte, 5-FU and 5-FU combination treatment groups. The proportion of PCNA- and Ki67-positive cells in the Arte and 5-FU combined treatment group significantly decreased. The 5-FU group showed a slight increase in the proportion of cells positively stained for the apoptosis indicator Cleaved-caspase-3. The number of Cleaved-caspase-3-postive cells in the Arte + 5-FU group was significantly increased compared to that in the 5-FU treatment group. Bcl-2 suppresses apoptosis and is an indicator of apoptosis inhibition [25]. There was no statistically significant reduction in Bcl-2 levels in the 5-FU group compared to the control group and the Arte group, but the Arte + 5-FU group showed a more pronounced reduction in Bcl-2 levels. The above results show that Arte significantly enhanced the antitumor sensitivity of 5-FU, slowed the growth of malignant cells, and increased the apoptosis tumor cells.

## Discussion

5-Fluorouracil can induce intestinal damage and even affect patient quality of life and CRC treatment effectiveness, as well as increase hospital costs and place more financial pressure on patients. There are currently no effective treatments for the side effects of 5-FU administration. For this reason, it is imperative to develop new and effective drugs to treat chemotherapy-induced side effects. In the current study, we sought to investigate the relationship between 5-FU and intestinal damage. Using HIEC cells and a BALB/c mouse model, our study revealed that intestinal inflammation and intestinal epithelial senescence cell accumulation were the two major mechanisms by which 5-FU causes diarrhea. In our experiments, we also discovered that Arte not only ameliorated 5-FU-induced intestinal damage but also increased the antitumor effectiveness of 5-FU without exacerbating the side effects.

Previous research by our team has shown that 5-FU can cause cell senescence [26, 27]. Although 5-FU has numerous clinical benefits, our data show that mice fed with 5-FU experience an increase in diarrhea rating indices, a reduction in body weight and a reduction in food and water intake. The results are consistent with previous findings [28, 29]. According to our findings, intestinal injury is mainly caused by intestinal inflammation, destruction of intestinal villi and senescent cell accumulation. In BALB/c mice with diarrhea, 5-FU treatment caused cellular senescence in the colonic epithelium, short intestinal villi, and irregular colonic structures. The cellular senescence in the colon was significantly reduced and the intestinal villi were intact in the Arte group. We monitored the indices of mice, and diarrhea, water intake, and body weight all improved. In addition, Arte reduced p53 and p21 mRNA expression levels in colon tissue. According to our study, Arte could provide relief from 5-FU-induced intestinal damage.

Growing evidence indicates that activated mTOR plays a pivotal role in both senescence and intestinal inflammation. Yangzhou et al. reported that PIN1 protects against senescence by inhibiting the mTOR pathway [30]. Naser-Aldin Lashgari and his team collected data from clinical sources, and the results showed that inhibition of the mTOR signaling pathway reduces inflammation and cytokines in inflammatory bowel disease (IBD) [31]. According to our study, 5-FU induced activation of the mTOR signaling pathway, while Arte inhibited mTOR, thus delaying aging and inflammation. The above literature shows that the mTOR signaling pathway regulation is an important mechanism by which 5-FU causes intestinal damage. Further studies will be required to prove that mTOR inhibitors can also prevent 5-FU-induced cell aging. several literatures also report that mTOR inhibitors can enhance the anti-colorectal cancer activity of 5-FU [32, 33]. Moreover, there is still work to be done to determine the precise antitumor mechanism and whether inhibiting the mTOR signal pathway might reduce chemotherapy side effects.

NF-kappa B (NF-κB) plays a crucial role in cell adhesion, proliferation, differentiation, and apoptosis defense, along with the inflammatory response and host immunological response, and is a dimeric transcription factor [34, 35]. IL-1β receptors and TNF-α receptors can activate canonical NF-κB signaling, which is necessary for initiating inflammatory responses [36]. p38MAPK is one of the four principal mitogen-activated protein kinases (MAPK) cascades and was discovered first in studies on endotoxin-induced cytokine expression. It has been reported to be involved in inflammation, cell growth, differentiation, cell death, and the cell cycle [37, 38]. In our study, 5-FU activated the NF-kB and p38MAPK (p65, p38) signaling pathways in vivo and in vitro, whereas Arte inhibited the protein phosphorylation levels of p65/p38, as well as the levels of IL-6, TNF-α, and IL-1β. Furthermore, HE staining yielded consistent results in BALB/c mice, and Arte relieved intestinal inflammation. As a result of the current study, Arte appears to protect against enteritis and reduce NF-κB p65 levels as well as p38MAPK expression levels. Drugs inhibiting the NF-kB p65 and p38MAPK signaling pathways may be future treatments for enteritis.

To our knowledge, this is the first study examining the antitumor effects of Arte combined with 5-FU. We investigated the chemosensitivity effects of the combination treatment on HCT116 cells. When combined with 5-FU therapy, Arte can be an effective sensitizer. HCT116 cells yielded consistent results from both CCK8 experiments that the antitumor effect of Arte combined with 5-FU was greater than that of 5-FU alone. In HCT116 xenografts, Arte added to 5-FU also increased the chemotherapy response, consistent with the cell results. We monitored the safety of drug therapy in experimental model animals. BALB/c nude mice lost a significant amount of weight after treatment with 5-FU, while the 5-FU + Arte mice showed no weight loss. In terms of antitumor effects, compared to the 5-FU group, the 5-FU + Arte group had obvious antitumor effects. In addition, tumor HE staining and immunohistochemical indices (PCNA, Ki-67, Cleaved-caspase-3, Bcl-2) confirmed that Arte could be a relatively effective sensitizer and enhance 5-FU antitumor activity.

In summary, in our study, we first found that Arte alleviates HUVEC, HIEC, and intestinal epithelial senescence and inflammation caused by 5-FU, which was one of the main mechanisms by which Arte relieves the intestinal damage induced by 5-FU. Second, our study examined the antitumor efficacy of 5-FU in combination with Arte in CRC cells. We discovered that Arte improved the anticancer efficacy of 5-FU when used in a BALB/c nude xenograft tumor model. Our research provides a new theoretical basis for developing novel therapeutic agents and regimens for clinical patients.

## Data Availability

The datasets generated and/or analyzed during the current study are available from the corresponding author on reasonable request.

## Abbreviations

Arte: Artesunate
AMPK: Adenosine 5′monophosphate-activated protein kinase
CRC: Colorectal cancer
D: Day
5-FU: 5-Fluorouracil
FUICM: 5-FU-induced colonic mucositis
HUVEC: Human umbilical vein endothelial cell
HIEC: Human intestinal epithelial cells
IECs: Intestinal epithelial cells
Ins: Insulin
IBD: Inflammatory bowel disease
mTOR: Mammalian target of rapamycin
MAPK: Mitogen-activated protein kinase
p38MAPK: P38 Mitogen-activated protein kinase
PI3K: Phosphatidylinositol-3 kinases
ROS: Reactive oxygen species
SASP: Senescence-associated secretory phenotype
SIPS: Stress-induced premature aging
TNF-α: Tumor necrosis factor-alpha
NF-kappaB: Nuclear factor-kappa B
